# Shot Noise in a Metal Close to the Mott Transition

**DOI:** 10.1021/acs.nanolett.4c02521

**Published:** 2024-12-09

**Authors:** Yiou Zhang, Shashi Pandey, Sergei Ivanov, Jian Liu, Sergei Urazhdin

**Affiliations:** † Department of Physics, Emory University, Atlanta, Georgia 30322, United States; ‡ Department of Physics and Astronomy, University of Tennessee, Knoxville, Tennessee 37996, United States

**Keywords:** Mott transition, electron correlation, shot noise, spin−orbit coupling

## Abstract

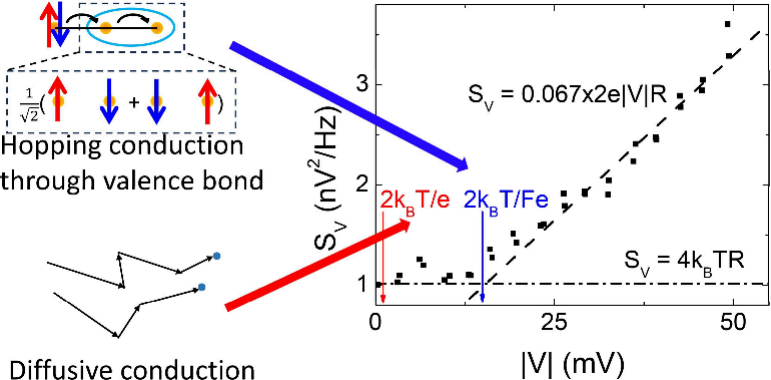

SrIrO_3_ is a metallic complex oxide with unusual electronic and magnetic properties believed to originate from electron correlations due to its proximity to the Mott metal–insulator transition. However, the nature of its electronic state and the mechanism of metallic conduction remain poorly understood. We demonstrate that the shot noise produced by nanoscale SrIrO_3_ junctions is strongly suppressed, inconsistent with diffusive quasiparticle transport. Analysis of thermal effects and scaling with the junction length reveals that conduction is mediated by collective hopping of electrons almost localized by correlations. Our results provide insight into the non-Fermi liquid state close to the Mott transition and advance shot noise measurements as a powerful technique for the study of quantum materials.

Interplay among crystal field effects, electron interactions, and spin–orbit coupling (SOC) leads to rich phenomenology of the quantum states of matter such as topological Mott insulators,^1^ unconventional superconductors,^2^ and quantum spin and anomalous Hall effect insulators.^3,4^ Complex oxides are archetypal quantum materials whose properties are tunable by varying composition and doping, serving as a test bed for the underlying mechanisms.^5^ A Ruddlesden–Popper series of iridium oxides Sr_*n*+1_Ir_*n*_O_3*n*+1_ provides a unique insight into the effects of dimensionality and large SOC on electron correlations. The 5*d* levels of Ir are split by the crystal field and SOC, resulting in a half-filled spin–orbit coupled *J*_*eff*_ = 1/2 valence state whose properties can be approximated by the Mott–Hubbard model of metal–insulator transition (MIT).^6−8^ According to this model, the MIT is controlled by the ratio *W*/*U* of the effective bandwidth *W* to the Mott interaction *U*.^7−9^ In iridates, slabs of *n* perovskite layers are separated by the “blocking” SrO layers, allowing effective bandwidth tuning. Indeed, quasi-2D Sr_2_IrO_4_ (*n* = 1) and Sr_3_Ir_2_O_7_ (*n* = 2) are Mott insulators, while SIO (*n* = *∞*) is a conducting material that, based on angular-resolved photoemission spectroscopy (ARPES) and *ab initio* calculations, is believed to be a semimetal.^7−11^ Thus, SIO is a metal (or a semimetal) close to the MIT, in the sense that the Mott criterion for electron localization is almost satisfied.

Proximity to the Mott transition is evidenced by the anomalous transport properties of SIO films. The resistivity ρ of thick films is typically almost independent of temperature *T*. In thin films, it starts to increase with decreasing *T*.^10,12^ Ultrathin films exhibit an MIT, consistent with the effects of reduced dimensionality in quasi-2D iridates.^11,13,14^ Transport properties of moderately thin SIO films were interpreted in terms of weak localization,^11,15,16^ and those of ultrathin films, in terms of variable-range-hopping (VRH) and strong (Anderson) localization due to disorder.^11,13,17^ However, the dependence of effective VRH dimensionality on film thickness^17^ and inconsistencies between the effects of strain and film thickness on magnetoresistance^11^ indicate significant electron correlations not captured by these models. Indeed, observations of magnetism in thin SIO films and at the interfaces with ferromagnets were interpreted as evidence for significant Mott correlations.^18−20^ Meanwhile, electronic Raman spectroscopy shows a spectral continuum similar to that in superconducting cuprates, inconsistent with the single-particle Fermi liquid picture.^21^

To elucidate the nature of the electronic state in SIO, we performed measurements of shot noise (SN): white noise produced by biased junctions due to the discrete nature of charge carriers. The current noise in tunnel junctions is^22^
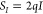
1where *q* is the effective carrier charge and *I* is the bias current. The Fano factor  provides a unique probe for electron correlations, as demonstrated for Cooper pairing in superconductors^23,24^ and charge fractionalization in quantum Hall systems.^25,26^ SN is suppressed in mesoscopic conductors. For diffusive single-electron transport in Fermi liquids, the Fano factor is reduced to 1/3 if electron interaction is weak, and  if electrons are thermalized by interactions.^27,28^ The value of *F* is independent of the conductor length *L* if the latter is smaller than the electron–phonon scattering length, *l*_*e*–*ph*_, and becomes suppressed by electron–phonon interaction in longer junctions.^29^

Strong suppression of SN in a non-Fermi liquid “strange” metal was attributed to the lack of well-defined quasiparticles.^30^ Since the “strange” metal state is often observed close to the Mott MIT, SIO may be conjectured to also exhibit anomalous SN. Here, we report strong SN suppression in SIO junctions, in agreement with the main result of ref (30). Analysis of thermal effects and scaling with *L* allows us to eliminate electron–phonon scattering as the SN suppression mechanism and shows that charge transport is mediated not by quasiparticle diffusion but by correlated hopping of electrons almost localized by interactions. Our results provide insight into the nature of the non-Fermi liquid state close to the Mott MIT.

The studied nanostructures were patterned from two films: an SIO(20) film grown on (001)-oriented single-crystalline TbScO_3_ and a SIO(35) film grown on (001)-oriented SrTiO_3_. Numbers in parentheses are thicknesses in nanometers. The consistency of results for two different substrates and film thicknesses confirms their generality. The films were deposited from a SrIrO_3_ target by pulsed laser deposition, with the substrate temperature maintained at 700 °C, the KrF excimer laser fluence of 2 J/cm^2^, and oxygen pressure of 0.1 mbar. The growth was monitored by *in situ* reflection with high-energy electron diffraction. The high quality of epitaxy was confirmed by the sharp peaks in the X-ray diffraction measurements, Figure 1(a) (see also Supporting Information). Pronounced Kiessig fringes in low-angle reflectivity indicate smooth film interfaces (see the inset in Figure 1(a)).

**Figure 1:**
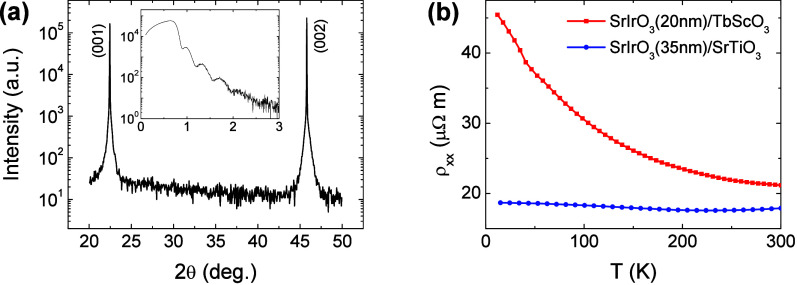
(a) X-ray diffraction pattern around the TbScO_3_ (001) and (002) reflections for the SIO(20 nm)/TbScO_3_ film. The film peaks are overlapping with the substrate because of the similar pseudocubic lattice parameters between SIO and TbScO_3_. The inset shows Kiessig fringes in low-angle X-ray reflectivity, demonstrating low surface/interface roughness. (b) Resistivity vs temperature for unpatterned SrIrO_3_(20)/TbScO_3_ and SrIrO_3_(35)/SrTiO_3_ films.

The resistivity ρ(*T*) of SIO(20) on TbScO_3_ exhibits a modest nondiverging increase with decreasing *T*, Figure 1(b). Meanwhile, for SIO(35) on SrTiO_3_, ρ is smaller and almost independent of *T*, which can be attributed to hopping enhancement (larger effective *W*) due to compressive strain and larger film thickness.^16^ Moderate differences in resistivity and its temperature dependence, compared with orders of magnitude for SIO films with larger thickness differences,^10,31^ suggest that the transport mechanisms in the two films are similar, as confirmed by the SN measurements discussed below.

The films were patterned into nanoscale metallic junctions using multistep e-beam lithography (see Supporting Information for details). Junctions with length *L* = 100–500 nm were lithographically defined strips, Figure 2(c). Shorter junctions were fabricated by using two different approaches. In a vertical nanocontact (VNC) with a size of 40 nm (Figure 2(a)), current confinement defined junction length close to its diameter. In an alternative approach, break junctions with length *L* ≈ 50 nm were formed by electromigration starting with two connected sharp-point electrodes (Figure 2(b)). SN in VNCs may be underestimated due to the resistance of the bottom SIO lead, while break junctions are prone to large geometric uncertainty. Nevertheless, the consistency of results for two different junction geometries supports their validity. The carrier mean free path (mfp) estimated from Hall measurements (see Supporting Information for details) is much smaller than the length of all the studied metallic junctions, excluding the existence of high-transmission quantum channels. The short mfp has also been confirmed in similar films by magnetoresistance measurements.^11^

**Figure 2:**
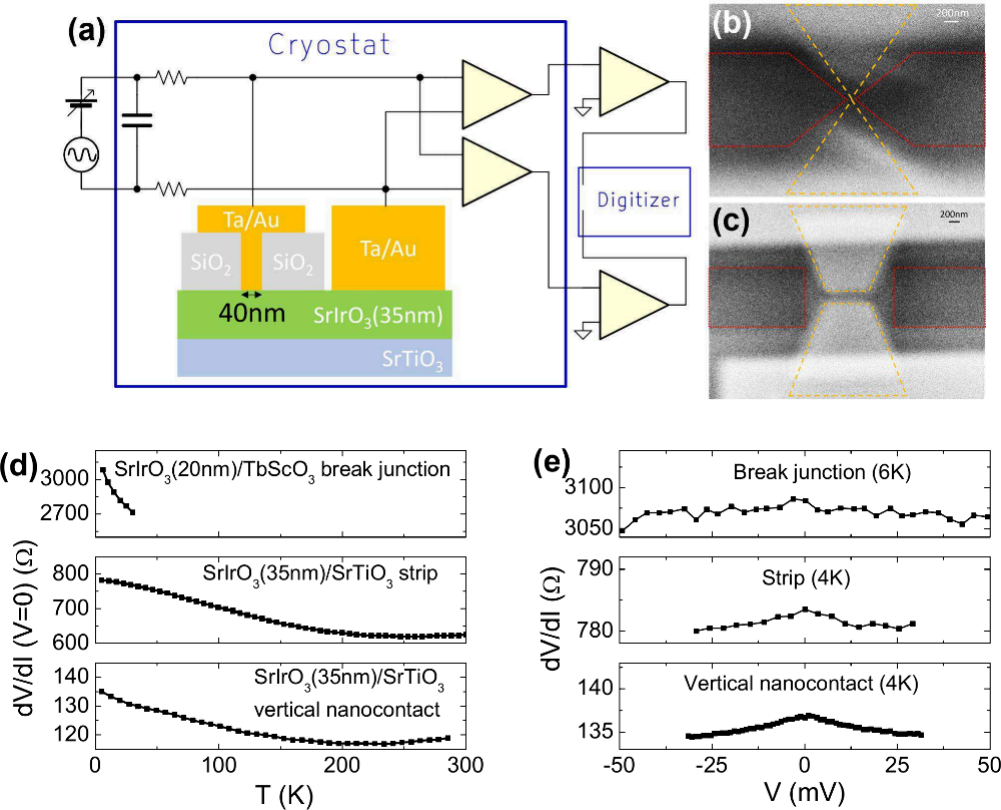
(a) Schematic of the vertical nanocontact and measurement circuit. Variable DC and AC (100 μV at 100 Hz) voltage for resistance and SN measurements was sourced through two 12 kΩ resistors. Noise was measured via two stages of parallel amplification (cryogenic first stage) followed by signal cross-correlation. (b, c) SEM images of a break junction (b) and a 100 nm long, 400 nm wide strip (c). Yellow lines outline electrodes; red shows regions of SIO removed by Ar ion milling. (d) Device resistance vs temperature. (e) Device resistance vs bias voltage, at the lowest measurement temperature.

The resistance *R*(*T*) of the junctions was consistent with ρ(*T*) for unpatterned films, showing that the electronic properties of SIO were not compromised by nanopatterning and the contribution of contact resistance was insignificant. For break junctions, applying bias at high temperature causes slow drift of device resistance, probably due to thermally activated migration of Au atoms. To ensure stability of the break junctions over the whole measurement process, bias was applied only at cryogenic temperatures, *T* < 30 K. Ohmic properties of devices were tested by the dependence of *R* on bias. Resistance was almost constant for the break junctions and slightly decreased with bias for other structures, Figure 2(e). It is unlikely that the decrease is caused by a Schottky contact barrier, which would have been larger in break junctions based on the SIO(20) film since it is closer to the insulating state, and due to the small effective contact area. Regardless of the origin, the variation of *R* is too small for the underlying transport process to significantly contribute to SN.

Our central result is the anomalous SN produced by the SIO nanojunctions under bias. Noise measurements were performed using the cross-correlation technique, Figure 2(a) (see Supporting Information for details). The technique was validated by measurements of thermal (Johnson) noise versus *T*, which were in agreement with calculations without any adjustable parameters, Figure 3(a). Noise measurements on a control gold nanowire sample (Supporting Information) also give shot noise as predicted in the single-particle Fermi liquid picture, providing an independent validation of our measurement approach. Electromagnetic interference (EMI) was manifested by sharp peaks and low-frequency noise in the noise spectra, which were the largest in the break junctions [inset in Figure 3(b)]. A frequency window where the detected noise was white and free from EMI was used in the measurements.

**Figure 3:**
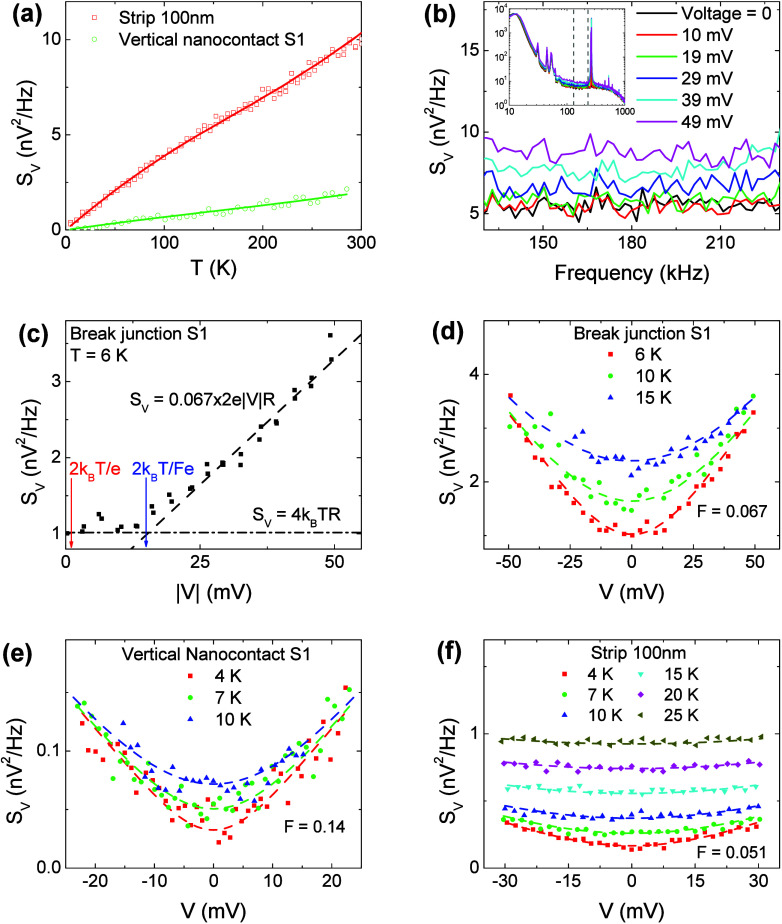
(a) Johnson noise vs *T* for two SIO junctions. The solid lines are the expected values *S*_*V*_ = 4*k*_*B*_*TR*(*T*). The nonlinearity is due to resistance variation with temperature; see Figure 2(e). (b) Noise spectra of a break junction at different bias. Inset: broadband spectra showing EMI peaks and SN detection window. (c) *S*_*V*_ vs |*V*| for a break junction at *T* = 6 K. The slope of the linear dependence of *S*_*V*_ on *V* at large bias gives *F* = 0.067. The crossover between Johnson noise and SN is . (d–f) *S*_*V*_ vs *V* for three different junctions. The dashed curves are fittings with eq 3.

Noise increased with bias while remaining white, as expected for SN and eliminating possible artifacts from flicker noise, Figure 3(b). The dependence of voltage noise *S*_*V*_ on the bias magnitude |*V*| for one of the two studied break junctions is shown in Figure 3(c). Johnson noise dominates at a small *V*, resulting in almost constant *S*_*V*_ = 4*k*_*B*_*TR*. SN is manifested by the linear dependence at large *V*, which can be described by
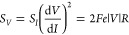
2The Fano factor *F* = 0.067 is much smaller than 1/3 expected for weak electron–electron interaction^27−29,32^ or  for electrons thermalized by interactions, consistent with SN suppression in a “strange” metal.^30^ Electron–phonon interaction would result in a nonlinear dependence ,^27,28^ which is not observed. Extra noise due to junction heating is ruled out by the estimates based on the dependence of resistance on bias and analysis of heat diffusion including both electron–electron and electron–phonon scattering (see Supporting Information for details). The negligible role of thermal phonons is also confirmed by the lack of temperature dependence discussed below. Therefore, in the single-particle Fermi liquid picture, suppressed SN cannot be explained by either electron–phonon or electron–electron interaction.

Analysis of the thermal effects provides insight into the mechanism of SN suppression. The crossover bias *V*_*th*_ from Johnson noise to SN is defined by the intersection between the extrapolation of the linear dependence for SN and bias-independent Johnson noise, Figure 3(c). In the Landauer approximation,  is expected from the analysis of the Fermi–Dirac distribution in electrodes.^33^ The same result is obtained in the diffusive approximation^27,28^ from the analysis of thermal distribution in the junction. Instead, the observed thermal broadening is close to , 15 times larger than expected from these theories.

An alternative model for electron transport is hopping through localized states in materials close to the insulating state^34−37^ or their sequential tunneling through defective barriers.^38,39^ If the transmission process comprises *N* hopping segments or tunneling events, the average bias voltage per segment is *V*/*N*, resulting in the Fano factor *F* = 1/*N* and thermal broadening , consistent with our data. By comparing fits obtained with several functional forms (see Supporting Information for details), we found that the entire dependence of noise on bias was best described by
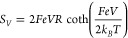
3, consistent with hopping transport.

Figure 3(d–f) shows *S*_*V*_ vs bias for all three studied junction types. Despite different junction geometries, lengths, and values of *F*, eq 3 provided a good fit for all the junctions, with the effect of thermal broadening well-captured for all measurement temperatures (see Supporting Information for additional data and fitting).

Figure 4(a) shows the temperature dependence of the Fano factors extracted from fitting the noise data to eq 3. For clarity, only the three junctions shown in Figure 3(d–f) are included. In the measured temperature range, the value of *F* is constant for all of the junctions within the fitting uncertainty. One could hypothesize that SN suppression is caused by scattering with thermally excited bosons (either phonons or bosonic modes of interacting electrons) that freeze out at a low *T* below the measured range. In this scenario, we would have observed an increase of SN suppression with temperature due to the higher boson population, as expected from Bose–Einstein statistics. One would also expect such a freeze-out to be manifested in the variation of electronic transport properties, which is not observed in similar SIO films down to 100 mK.^10,11^

**Figure 4:**
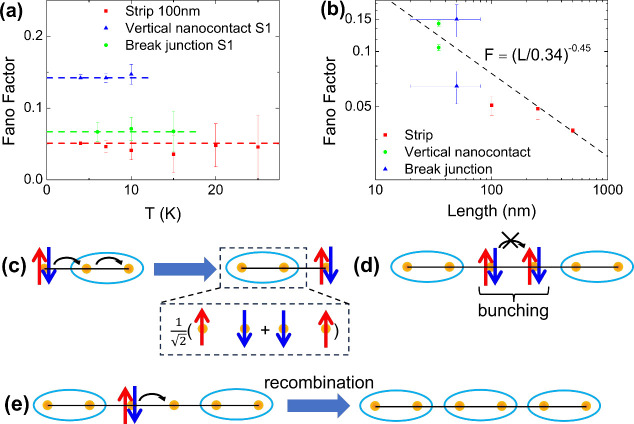
(a) Fano factors vs temperature for three junctions with different geometries. The dashed horizontal lines are guides to the eye. (b) Fano factors of all the measured junctions as a function of the junction length. Uncertainty of *F* is estimated from numerical fitting, and uncertainty of the junction length, from the SEM images and resistance. The dashed line shows the linear fitting of log(*F*) versus log(*L*), which gives  with *L*_*c*_ = 0.34 ± 0.05 nm and β = 0.45 ± 0.01. (c–e) Schematics of quasi-localized charge hopping in the VB state. (c) Hopping of an excess electron (two electrons on site) through the VB. (d) Hopping of an excess electron is blocked by another excess electron due to the Pauli exclusion principle, causing bunching of hopping events. (e) Recombination of an excess electron and a hole results in hopping blockage for other charges.

The mechanism of hopping is elucidated by the dependence of the Fano factor on the junction length *L*, as shown for all of the studied junctions in Figure 4(b). The value of *F* decreases with *L*, as expected due to the increasing number of hopping events. Linear fitting of log  *F* versus log  *L* gives , with the characteristic hopping length *L*_*c*_ = 0.34 ± 0.05 nm and exponent β = 0.45 ± 0.01. We note that the length dependence of the Fano factor may be somewhat different between the two films due to different film thickness and substrate. However, if break junctions on SIO(20)/TbScO_3_ are excluded (both VNCs and nanostrips are based on the same SIO(35)/SrTiO_3_), the parameters obtained from the fitting remain almost the same, *L*_*c*_^′^ = 0.33 ± 0.05 nm and β′ = 0.45 ± 0.01. This indicates that the scaling relationship is likely to be generic in moderately thin SIO.

The scaling with length is slower than *F* ∝ 1/*L* expected from independent charge hopping, while the hopping length close to the pseudocubic lattice constant *a* = 0.39 nm of SIO^10^ is unphysically small for single-particle hopping in a defect-dominated energy landscape. We note that both the scaling exponent and the hopping length may be prone to large errors due to the small number of data points and large uncertainties in junction lengths. Nevertheless, these uncertainties cannot account for the large discrepancies in the single-particle hopping picture. These anomalous behaviors can be explained by strong correlations, as discussed below.

The lack of low-temperature resistivity divergence inconsistent with VRH^40^ is commonly interpreted as evidence for diffusive charge transport in conducting SIO films. However, our SN measurements demonstrate that conduction in the studied SIO films is mediated by hopping. Different from typical hopping conductance, the resistivity of SIO does not exhibit an exponential dependence on either temperature or bias voltage, indicating that there is no minimum energy scale for hopping. This unconventional hopping mechanism is likely related to other electronic properties of SIO not described by the Fermi liquid picture and often attributed to correlation effects.^12,21,41^ We propose a qualitative mechanism for the correlated hopping in SIO based on the Hubbard model of a half-filled band with the effects of hopping *t* somewhat dominant over electron interaction *U*. This mechanism explains hopping conductance without resistivity divergence at low *T*, as well as the scaling relationship between the Fano factor and junction length. Because of the large measurement uncertainties, this agreement does not provide a definitive confirmation for the proposed mechanism. Nevertheless, our model suggests a framework for the future more detailed analyses.

If the hopping term *t* is somewhat dominant over electron interaction *U*, the insulating Mott state is suppressed, which may be facilitated by large SOC even if the Mott criterion for MIT is satisfied.^7^ Under these conditions, a Fermi liquid may not be the ground state because of the energy reduction provided by the local Mott singlet correlations, which can be described as incoherent Cooper pairs.^42^ In the resonating valence bond (RVB) model of the non-Fermi liquid state in doped Mott insulators, such pairs are approximated by valence bonds (VBs), singlets formed by electrons quasi-localized on neighboring sites.^43^ In the SIO, a relatively large ratio *t*/*U* allows significant local charge fluctuations. Since VBs constrain site occupancy to a single electron, a quasi-localized excess electron or a hole can be located only on sites that do not participate in VBs. Consequently, charge diffusion is prevented by the dense VBs that limit the possible charge positions.

To develop a qualitative picture of charge transport in this state, consider a minimal three-site model of an excess electron next to a VB (Figure 4(c). The electron can move by exchanging places with the VB via double hopping. The final state has the same energy as the initial state, since they differ only by the relative positions of electron and VBs. On the other hand, in the intermediate state with the extra electron on the middle site, nearest-neighbor singlet correlation is suppressed, resulting in a higher energy. Thus, this transport process is elastic electron tunneling governed by correlations, which in contrast to single-particle hopping or inelastic tunneling in a disordered energy landscape does not lead to resistivity divergence at low *T*.

The characteristic hopping length *L*_*c*_ = 0.34 nm determined from the scaling of the Fano factor with *L* is close to the pseudocubic lattice constant of 0.39 nm.^10^ This value would be unphysically short in the single-electron picture but is consistent with the proposed mechanism. The anomalously small scaling exponent can also be explained by correlations. A suppressed scaling exponent caused by electron bunching due to the Pauli exclusion principle was predicted in simulations of hopping in confined geometries.^34,37^ Similar bunching was observed in tunnel junctions as charge and spin blockage.^39,44−46^ In 2D systems, the effects of bunching are modest due to the existence of multiple hopping paths, resulting in the scaling exponents close to β = 1.^35,37^ On the other hand, strong bunching effects in 1D chains^34^ and quasi-1D systems are predicted to yield β = 0.5 remarkably close to *b* = 0.45 observed in our measurements.

In our model of correlations in SIO, charge hopping is expected to be confined to quasi-1D paths by the dense arrangement of VBs, which should lead to efficient hopping blockage due to excess charge bunching, Figure 4(d). Furthermore, overlap of electron and hole hopping paths can result in their annihilation (Figure 4(e)), creating a VB that reduces possible configurations of VBs that permit charge hopping. The latter mechanism may explain why the observed value β = 0.45 is even smaller than the value of 0.5 expected for 1D hopping of a single type of charge carriers. The scaling exponent may be also reduced by the strong correlations beyond the effective single-particle hopping picture.

The proposed model can explain the thickness dependence of the transport properties in moderately thin films. Increased geometric confinement at a smaller film thickness leads to larger overlap between charge hopping paths, increasing the probability of blocking via the mechanisms discussed above. In contrast to single charge swap with a VB, these processes involve interactions between excess charges and do not necessarily conserve energy, resulting in an increase of resistivity at low *T*.^10,47,48^ A similar mechanism can explain the evolution of electronic properties across the Sr_*n*+1_Ir_*n*_O_3*n*+1_ iridate family with *n* > 2, before the onset of the Mott state.

In summary, we studied shot noise in metallic SrIrO_3_ nanojunctions with lengths between 40 and 500 nm. All the studied junctions exhibit strong shot noise suppression which cannot be explained by electron–electron or electron–phonon interaction in the single-particle Fermi liquid picture. Analysis of thermal broadening of noise dependence on bias shows that conduction occurs not via the commonly assumed electron diffusion but rather by charge hopping. The dependence of shot noise on the junction length reveals the dominant role of electron correlations. We propose that the unusual electronic properties of SrIrO_3_ films can be explained by charge hopping along quasi-1D conduction paths confined by Mott singlet correlations. This mechanism lifts the constraints imposed by energy conservation on single-particle hopping, avoiding resistivity divergence at low temperatures. It should be noted that the proposed correlated hopping remains hypothetical due to the lack of direct evidence and the scaling relationship is not sufficiently precise to draw definitive conclusions about the underlying mechanism. Nevertheless, these uncertainties do not affect our main conclusion about the charge transport by hopping in SIO. Investigations of shot noise at lower temperatures or over a larger range of junction lengths may yield a more precise dependence of the Fano factor on length, providing further insight into the hopping mechanism.

The proposed mechanism may be relevant to one of the puzzling hallmark features of the “strange” metal state observed in other materials close to the Mott transition such as high-temperature superconductors: the nonsaturating linear temperature dependence of resistivity.^49^ A recently proposed theory explains SN suppression in a strange metal in terms of electron energy dissipation due to coupling to quantum-critical bosons.^50^ This picture based on diffusive transport is not directly applicable to SIO. Nevertheless, it is qualitatively consistent with our proposed model, as the dense VBs represent a simple model of a bosonic field formed by correlated electrons.

## Supporting Information Available

Additional X-ray measurement on SIO(35)/SrTiO_3_; experimental details on device fabrication and noise measurement; Hall and magnetoresistance measurement on SIO/SrTiO_3_ modeling and fitting of shot and Johnson noise; analysis of heating of nanojunctions under finite bias; noise measurement on additional SIO nanodevices and gold nanowire as a control sample (PDF)
