# Supplementary material for: Shot Noise in a Metal Close to the Mott Transition
Source: Nano Lett. 2024 Dec 9;24(50):15943–9. doi: 10.1021/acs.nanolett.4c02521 (PMC11660216; doi:10.1021/acs.nanolett.4c02521)
Supplement: Supplementary file 1 — nl4c02521_si_001.pdf [file nl4c02521_si_001.pdf]

Supplemental Materials: Shot noise in a metal close to Mott transition

Yiou Zhang,*,† Shashi Pandey,‡ Sergei Ivanov,† Jian Liu,‡ and Sergei Urazhdin†

†*Department of Physics, Emory University, Atlanta, Georgia 30322, USA*

‡*Department of Physics and Astronomy, University of Tennessee, Knoxville, Tennessee 37996, USA*

E-mail: yiou.zhang@emory.edu

X-ray measurement on $SrIrO_3$/$SrTiO_3$(001)

X-ray measurement on $SrIrO_3$/$SrTiO_3$(001) is shown in Fig.S1. Due to lattice mismatch between $SrIrO_3$ (SIO) and $SrTiO_3$ substrate, there is a clear distinction between the (001) and (002) diffraction peaks from SIO and $SrTiO_3$ substrate. The Kiessig fringes in low-angle X-ray reflectivity, shown in the inset of Fig.S1, demonstrate low surface/interface roughness similar to SIO/$TbScO_3$ as shown in Fig.1(a).

Device Fabrication

All the patterns are defined by electron beam lithography using MMA/PMMA bilayer resist. First, magnetron sputtering is used to deposit Ta(3)/Au(80) electrodes, which also serve as alignment markers. Here, thicknesses are in nanometers. Ar ion milling is then used to remove SIO in the areas between the electrodes, except for the regions where nanodevices

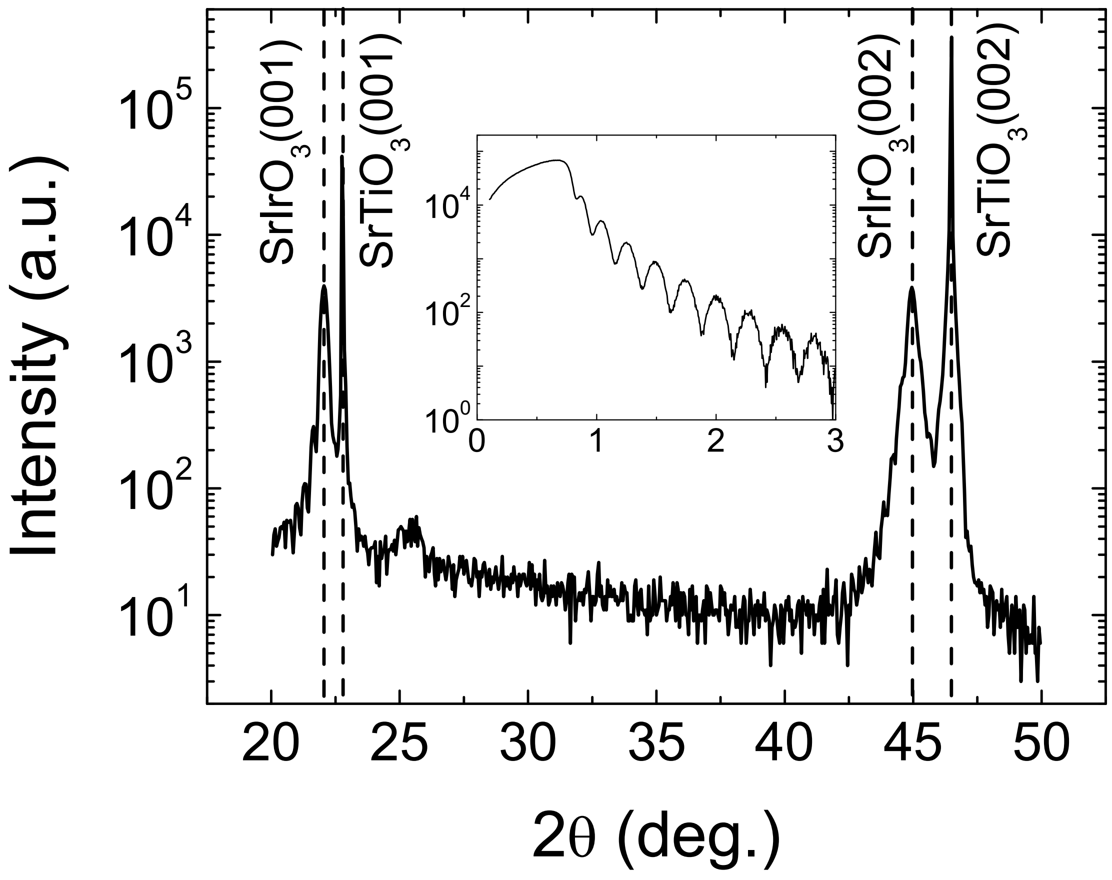

Figure S1: X-ray diffraction pattern of 35 nm $SrIrO_3$ film on $SrTiO_3$ substrate, showing clear (001) and (002) peaks from $SrIrO_3$ and $SrTiO_3$ substrate. The inset shows Kiessig fringes in low-angle X-ray reflectivity, demonstrating low surface/interface roughness.

are to be patterned. These patterns with leads serve as the common basis for all the nano-devices.

For nanostrips, two additonal Ti(3)/Au(50) electrodes separated by a narrow gap are thermally evaporated, each in contact with one of the large electrodes defined in the first step. The gap between the two nano-electrodes ranges from 100 nm to 500 nm, and their width at the gap is 4 times the gap size to obtain consistent device resistance of about $1\,k\Omega$. An additional Ar ion milling step is used to define the strip width, confining the current in the gap between the two nano-electrodes, and producing a well-defined device geometry.

Fabrication of break junctions is similar to that of strips, except that separation between the patterns in the polymer defining nano-electrodes is much smaller (about 20 nm), and the tips of the electrodes facing each other are shaped as sharp triangles. Tilted-angle thermal evaporation of Ti(1.5)/Au(15) is performed twice, with the sample tilted at $+15°$ and at $-15°$, respectively, with respect to the normal direction. Because of the undercut in the MMA/PMMA bilayer, the deposited electrodes' tips extend into the gap between the electrode shapes defined in the polymer. As a consequence, the two nano-electrodes touch at a narrow constriction, with resistance of $\sim 100\,\Omega$ between the electrodes. Pulses of voltage with gradually increasing amplitude are then applied to break the contact via Joule heating and electromigration, until resistance abruptly increases. The resulting resistance value $R \sim 1\,k\Omega$ is estimated to correspond to approximately 40 nm gap between the electrodes.

For vertical nanocontacts, a Pt(2)/Au(2) layer is first deposited by magnetron sputtering on top of the film with pre-patterned electrodes and ion milled SIO film, as described above. The Pt/Au layer serves as protection against diffusion of Al used in the next step as a mask. Al masks with diameter of 40 nm and thickness of 30 nm are then defined by electron beam lithography and thermal evaporation. Subsequently, Ar ion milling is used to remove Pt/Au on the SIO films, except for the area covered by Al, followed by deposition of insulating SiO_x in the same region by magnetron sputtering. Al mask is then removed by chemical etching in KOH solution, and Ta(3)/Au(40) top electrode is deposited by magnetron sputtering,

creating an electric contact to SIO only in the nanocontact opening not covered by SiO_x.

Measurements

All the noise and transport measurements are performed in a closed-cycle cryostat with 4 K base temperature. The measurement circuit is shown in Fig.S2. The DC bias and small AC excitation voltage (100 Hz) is produced by the analog and internal oscillator outputs, respectively, of the Signal Recovery 7265 lock-in amplifier. They are added, amplified and converted to a floating differential voltage by a pair of operational amplifiers. The single-ended-to-differential amplifier is followed by a RC low-pass filter (two $70\,\Omega$ resistors and one $8\,\mu F$ capacitor) and connected to device-under-test (DUT) through two large resistors ($12\,k\Omega$ each). One additional capacitor ($10\,nF$) is placed close to the large resistors and DUT, to filter out electromagnetic interference (EMI) from the biasing lines.

For noise measurement, the standard cross correlation method is implemented. First-stage amplifiers (Texas Instruments INA821) with gain of 28 are mounted inside the cryostat to reduce EMI. The amplified signals are further boosted by two additional amplification stages (both with Texas Instruments LTC6221) with the total gain of 320. The signals are then digitized by Measurement Computing USB-2020 digitizer at a sample rate of 3 MSa/s. The noise spectrum is obtained by averaging the cross-correlated spectrum 3000 times. The DC and low-frequency signal from one of the first-stage amplifiers is supplied to the lock-in amplifier through a buffer amplifier, which allows for simultaneous noise and resistance measurement. The frequency-dependent gain is calibrated by measuring Johnson noise of various resistors at different temperatures.

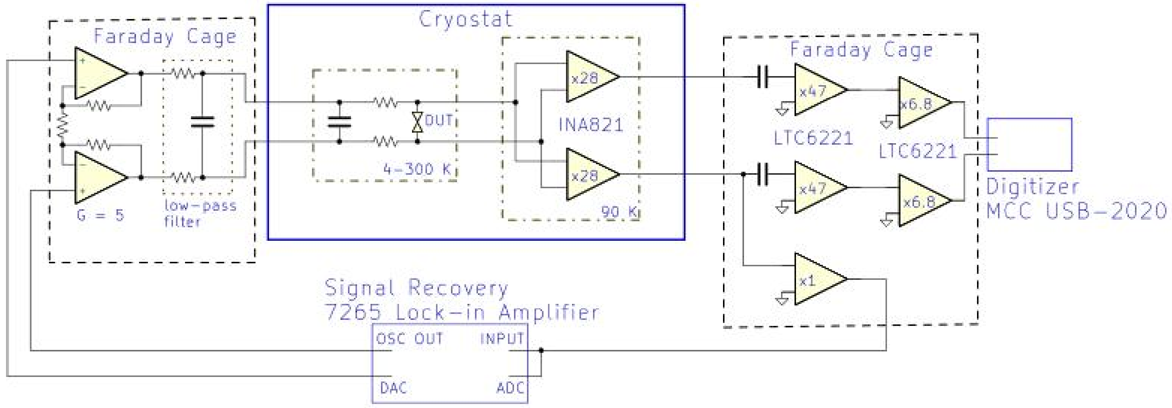

Figure S2: Circuit Schematic of the noise and transport measurement setup

Hall measurement and estimation of carrier mean free path

We have patterned a Hall bar structure on SIO(35)/$SrTiO_3$ using Ar ion milling to etch the SIO film into a standard Hall-bar shape and sputtered Ta(3)/Au(80) as the electrodes. The measured Hall coefficient, shown in Fig. S3, is consistent with previous work on similar films.[1] The temperature dependence of the Hall coefficient is attributed to the semi-metallic nature of SIO.

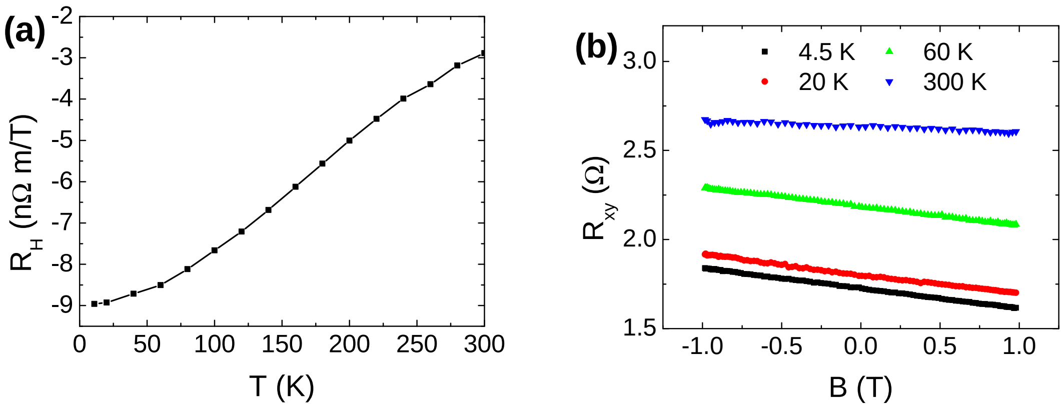

Figure S3: Hall measurement of SIO(35)/$SrTiO_3$. (a) Hall coefficient obtained from linear fitting of Hall resistance versus magnetic field. (b) Additional measurements over a larger field range to confirm the linearity of the Hall voltage.

As shown in the main text, transport in SIO is likely mediated by hopping rather than diffusion. Nevertheless, in this section we will use semi-classical transport theory to estimate mean free path (mfp) of the hypothetical carrier diffusion. According to the two-band Drude model, conductivity and Hall coefficient under small magnetic field are given by

$$\sigma = e(n_h\mu_h + n_e\mu_e) = en_e\mu_e(\frac{n_h\mu_h}{n_e\mu_e} + 1), \tag{S1}$$

and

$$R_H = \frac{n_h\mu_h^2 - n_e\mu_e^2}{e(n_h\mu_h + n_e\mu_e)^2} = \frac{1}{en_e}\frac{\frac{n_h\mu_h^2}{n_e\mu_e^2} - 1}{(\frac{n_h\mu_h}{n_e\mu_e} + 1)^2}, \tag{S2}$$

with n_e (n_h) and μ_e (μ_h) being the electron (hole) concentration and mobility respectively. The carrier mobility is defined as

$$\mu_{e/h} = \frac{e\tau_{e/h}}{m^*_{e/h}}, \tag{S3}$$

where τ_e (τ_h) and m^*_e (m^*_h) are the mean scattering time and effective mass of electrons (holes) respectively. As there are only two known values σ and R_H in Eq.S1 and Eq.S2, it is not possible to solve for four unknowns n_e, n_h, μ_e, and μ_h. Non-linear Hall effect measurement under very large magnetic field (over 15 T),[2] beyond our instrumental capabilities, would be essential. Nevertheless, n_e and μ_e can be estimated using reasonable assumptions for n_h/n_e and μ_h/μ_e. Following previous work,[2,3] we assume that $n_h = 1.5n_e$. Also, we assume $\tau_e = \tau_h = \tau$, so that the difference in mobility only comes from different effective mass ($m^*_e = 0.32m_e$ and $m^*_h = 1.58m_e$ as in Ref.,[2] which gives $\mu_h = 0.2\mu_e$). Note that the assumption of similar carrier scattering time is consistent with the picture of correlation-dominated hopping proposed in the main text. With these assumptions, we obtain

$$\mu_e = -\sigma R_H \frac{1 + \frac{n_h\mu_h}{n_e\mu_e}}{1 - \frac{n_h\mu_h^2}{n_e\mu_e^2}} = 6.55cm^2/(Vs), \tag{S4}$$

and

$$n_e = -\frac{1}{eR_H}\frac{1 - \frac{n_h\mu_h^2}{n_e\mu_e^2}}{(1 + \frac{n_h\mu_h}{n_e\mu_e})^2} = 4.1 \times 10^{20}cm^{-3}, \tag{S5}$$

where the resistivity ($19\mu\Omega m$) and the Hall coefficient ($-9n\Omega m/T$) for SIO(35) at cryogenic temperature was used. The obtained carrier concentration is slightly larger than previously reported values.[2,3] From carrier mobility, we can obtain the mean scattering time

$$\tau = \frac{m^*_e\mu_e}{e} = 1.2fs. \tag{S6}$$

To calculate mfp from mean scattering time, it is essential to know the Fermi velocity v_F. Owing to the complex electronic structure, the Fermi velocity v_F obtained from soft-x-ray

angle-resolved photoemission spectroscopy (SX-ARPES)[4] is highly anisotrpic. Also, it is generally different for electrons and holes. Nevertheless, an upper bound of v_F can be determined from the largest value ($5 \times 10^5 m/s$) along high-symmetry lines of the Brillouin zone. Using this value, we estimate mfp $l = v_F \tau \approx 1$ nm close to the lattice constant of SIO, which is typical for the Ioffe-Regel limit close to metal-insulator transition. A very short mfp is consistent with the analysis of shot noise described in the main text. Nevertheless, we emphasize that our estimate of mfp is based on multiple approximations. To obtain a more precise value one would need to perform additional characterization of electronic structure by advanced spectroscopic techniques, beyond the scope of this work.

On the one hand, the mfp is much smaller than any junction length of the studied nano-devices, excluding the presence of high-transmission quantum channels that could also potentially lead to shot noise suppression. On the other hand, the inferred value of mfp comparable to the lattice constant means that the semi-classical transport theory is not applicable, as has been extensively discussed for materials in the Ioffe-Regel limit. This inconsistency with the diffusive transport picture, together with the lack of low temperature resistance divergence expected for single-electron hopping, implies that exotic transport mechanism is present even in moderately thin SIO.

Magnetoresistance measurement of $SrIrO_3$

We have measured magnetoresistance of SIO(35)/$SrTiO_3$ using the Hall bar structure as described in the previous section, with magnetic field applied in the out-of-plane direction. As shown in Fig. S4, a very small positive MR ($< 0.1\%$) is observed at $T = 4.5$ K at fields up to 1 T, and drops to 0.01% at 20 K. At higher temperature MR becomes too small to be measured. The observed weak antilocalization is in qualitative agreement with previous work,[5] whereas much larger magnetic field and lower temperature is essential to obtain a more accurate value of magnetoresistance.

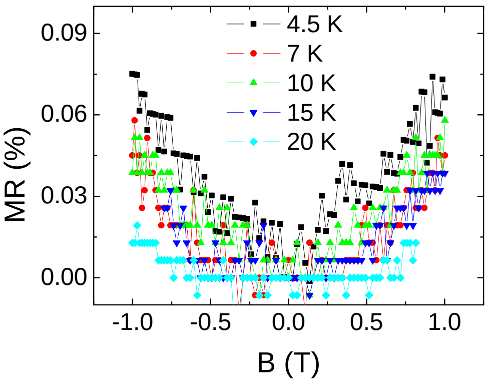

Figure S4: Magnetoresistance of SIO(35) under out-of-plane magnetic field.

Comparison of fitting with different approximations for shot and Johnson noise

For sequential tunneling and hopping, the total shot noise (SN) can be determined as a sum of noise from all individual tunneling/hopping events, taking into account that all the events are independent. The voltage noise S_V^i from each individual event can be expressed as

$$S_V^i = 2qV_iR_i \coth(\frac{qV_i}{2k_BT}), \tag{S7}$$

where q is the effective charge of the tunneling/hopping particles and V_i is the bias voltage. For simplicity, hereafter we assume that $\frac{\mathrm{d}V_i}{\mathrm{d}I_i} \cong \frac{V_i}{I_i} \equiv R_i$. Assuming that the tunneling/hopping events are equivalent, we obtain $V_i = \frac{V}{N}$ and $R_i = \frac{R}{N}$ where V is the total bias voltage, R is the total resistance, and N is the total number of tunneling/hopping events. If all the tunneling/hopping events are independent, their noise power adds, giving the total noise as

$$S_V = NS_V^i = \frac{2qV}{N}R\coth(\frac{qV}{2Nk_BT}) = 2FeVR\coth(\frac{FeV}{2k_BT}) \tag{S8}$$

with the Fano factor $F = \frac{q}{eN}$. Single-electron hopping/tunneling gives $F = 1/N$, while electron pairing would give $q = 2e$ and thus $F = 2/N$. To summarize this analysis, for N

independent noise sources in series, the total noise is $1/N$ times smaller than a single noise source with the same resistance and Fano factor, due to a combination of several scaling effects: i) the bias across each source scales as $1/N$, ii) the resistance of each source scales as $1/N$, and iii) the total noise is the sum of contributions from each source, resulting in an $\propto N$ scaling. Altogether, these contribution give $N/N^2 = 1/N$ noise scaling.

For diffusive transport in a single-particle Fermi liquid, the combined shot and Johnson noise is predicted to be described by

$$S_V = \frac{2}{3}(eVR\coth(\frac{eV}{2k_BT}) + 4k_BTR), \tag{S9}$$

which gives a reduced $F = 1/3$, as compared to tunnel junctions. Equation (S9) is obtained from both the random matrix approximation[6] and semi-classcial Boltzmann equation,[7,8] and remains valid for weak electron-electron and electron-phonon interaction. However, it cannot be used to fit noise characterized by Fano factor smaller than 1/3 as there is no free parameter in the equation. Ref. 9 utilized a modified form of Eq. (S9) with a smaller Fano factor

$$S_V = 2FeVR\coth(\frac{eV}{2k_BT}) + (1-F)4k_BTR. \tag{S10}$$

Another way to obtain reduced Fano factor is to consider a multistep transmission process, such as multiple tunneling/hopping, with every transmission characterized by a statistical distribution of transmission probabilities, as discussed in Ref. 6. This approach yields an equation Similar to Eq. (S8),

$$S_V = \frac{2}{3}\frac{eV}{N}R\coth(\frac{eV}{3Nk_BT}) + \frac{8}{3}k_BTR = 2FeVR\coth(\frac{3FeV}{2k_BT}) + \frac{8}{3}k_BTR, \tag{S11}$$

where $F = \frac{1}{3N}$. Eq.S8, Eq.S10, and Eq.S11 all give $S_V = 2FeVR$ for sufficiently large V and $S_V = 4k_BTR$ at zero bias. The major difference is the crossover between shot and Johnson noise, which is our main focus.

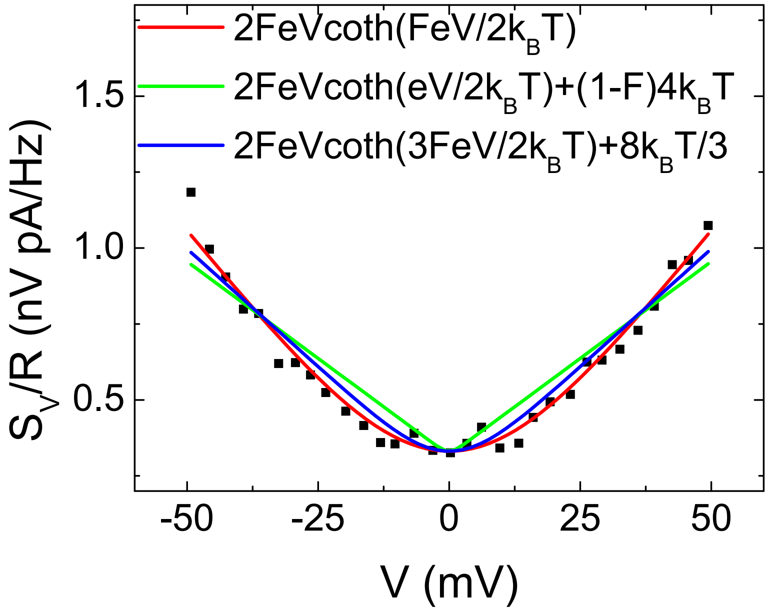

Figure S5: Numerical fitting of the measured noise shown in Fig.3(c) in the main text by three different equations.

To test how well these models describe shot noise in SIO, we numerically fit the measured noise from break junction S1, which is shown in Fig.3(c) in the main text. We fit $\frac{S_V}{R}$ instead of S_V, which accommodates different resistances of nano-devices. As can be seen in Fig.S5, Eq.S8 (red line) shows best fit to the measured noise, while Eq.S10 and Eq.S11 cannot fully capture the thermal broadening. Moreover, fitting by Eq.S10 and Eq.S11 underestimate shot noise under large bias. This shows that conductance in SIO is mediated by hopping instead of diffusion of charge carriers.

Heating effect on nanojunctions

Another possible source of extra noise is heating of the nanojunctions under bias voltage, which increases the Johnson noise. In this section we use phenomenological arguments and modelling to show that this mechanism cannot result the suppressed but still linear dependence of noise on bias observed in our measurements.

Since SIO exhibits a non-saturating resistance at low temperature, sample temperature can be estimated from the dependence of its resistance on bias, as shown in Fig.2(e) of the main text. It should be noted that there could be other mechanisms that cause the slight non-Ohmic behaviors in nanojunctions. Here, we assume that they are caused solely by the temperature increase to estimate an upper bound on heating effects. The Johnson noise of the nanojunctions can then be expressed as

$$S_V = 4k_B \int T\, dR \approx 4k_B T(V) R(V), \tag{S12}$$

where $T(V)$ is determined from resistance versus temperature measurement, and inhomogeneity of either temperature or resistivity is ignored.

We apply Eq. (S12) to break junction and VNC (noise data shown in Figs. 3(d) and (e) of the main text). For break junction (Fig.S6(a)), the extra noise obtained from Eq.S12 is negligible, as the estimated temperature increase is less than 1 K. While the estimated temperature increase is larger for VNC (7 K), Eq.S12 gives a smaller Fano factor at large bias and much smaller thermal broadening. Nevertheless, the effect of Joule heating should be comparable for break junctions and VNCs, due to the comparable length and effective constriction area. Since the break junctions on SIO(20) show a much larger temperature coefficient than VNCs on SIO(35), the resistance variation with bias should also be larger, contrary to our transport measurement. Therefore, based on the observed sample resistance variation, we can rule out a significant contribution of heating to the measured noise dependence on bias.

Alternatively, we can estimate the heating effects by modeling the temperature of the electron subsystem in SIO using the heat diffusion equation. Assuming that the electron subsystem is in local thermal equilibrium with local temperature $T_e(x)$ while the phonon subsystem remains at environment temperature ($T_{ph} = T_0$), we have[10]

$$\frac{d}{dx}(\kappa \frac{dT_e}{dx}) + \sigma E^2 - \Sigma(T_e^5 - T_0^5) = 0, \tag{S13}$$

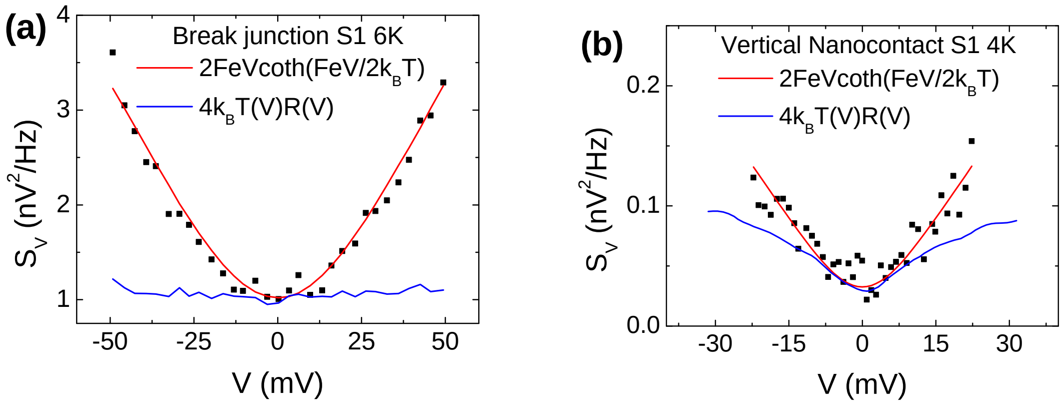

Figure S6: Comparison of shot noise estimated by hopping conduction (Eq.S8) and temperature increase (Eq.S12), for (a) break junction and (b) VNC shown in the maintext.

where σ is the electric and κ is the thermal conductivity of electron subsystem, E is electric field, and Σ characterizes electron-phonon coupling. For SIO nanojunctions, we neglect the temperature dependence of electric conductivity, $\sigma \approx const$, which for the uniform junction cross-section gives $E \approx (V/L)^2$. By setting $x' = x/L$, we can rewrite Eq.S13 as

$$\frac{d}{dx'}(\kappa\frac{dT_e}{dx'}) + \sigma V^2 - \Sigma L^2(T_e^5 - T_0^5) = 0, \tag{S14}$$

and

$$S_V = \frac{4k_B}{\sigma}\int_{-1/2}^{1/2} T_e dx'. \tag{S15}$$

If electron-phonon coupling is small, a linear dependence of the average temperature (and thus extra noise) on bias voltage can be obtained from Wiedemann-Franz law ($\kappa = \mathcal{L}\sigma T$), where L is Lorenz number. Equation (S14) is then reduced to

$$\frac{\mathcal{L}}{2}\frac{d^2T_e^2}{dx'^2} = -V^2, \tag{S16}$$

which gives $T_e \propto V$ at large bias and the Fano factor[7,8]

$$F = \frac{\pi k_B}{4e\sqrt{\mathcal{L}}} = \frac{\sqrt{3}}{4}. \tag{S17}$$

This value is significantly larger than observed in our measurements, and cannot explain the observed dependence on junction length.

Including electron-phonon scattering, Eq. (S14) becomes

$$\frac{\mathcal{L}}{2}\frac{d^2T_e^2}{dx'^2} + V^2 - \frac{\Sigma L^2}{\sigma}(T_e^5 - T_0^5) = 0, \tag{S18}$$

where the last term describing electron-phonon coupling term can result in reduced T_e and account for the length dependence. However, if the last term (dissipation through electron-phonon scattering) provides a non-negligible contribution, at large bias it becomes dominant over the first term (dissipation through electron diffusion), which leads to downcurving of noise as shown in Fig.S7. Such downcurvig is not observed in any of the studied nanojunctions. Moreover, the contribution of the electron-phonon coupling term should be temperature-dependent, contradicting the temperature-independent Fano factors in the studied SIO nanojunctions. Therefore, modeling by heat diffusion equation also does not support heating effect as the cause of suppressed but still linear noise dependence on temperature.

Noise measurement on additional nano-devices

Noise measurement on four additional nano-devices is shown in Fig.S8. For the vertical nanocontact S2 shown in Fig.S8(d), the sharp peaks at bias around $\pm 10mV$ originate from the $1/f$ noise.

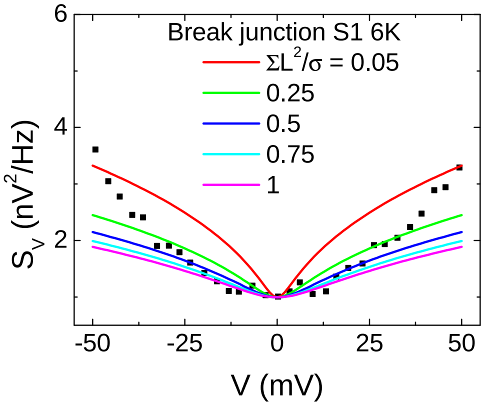

Figure S7: Fitting of shot noise of break junction by Eq.S18 with $\kappa = \mathcal{L}\sigma T$ and different values of $\Sigma L^2/\sigma$.

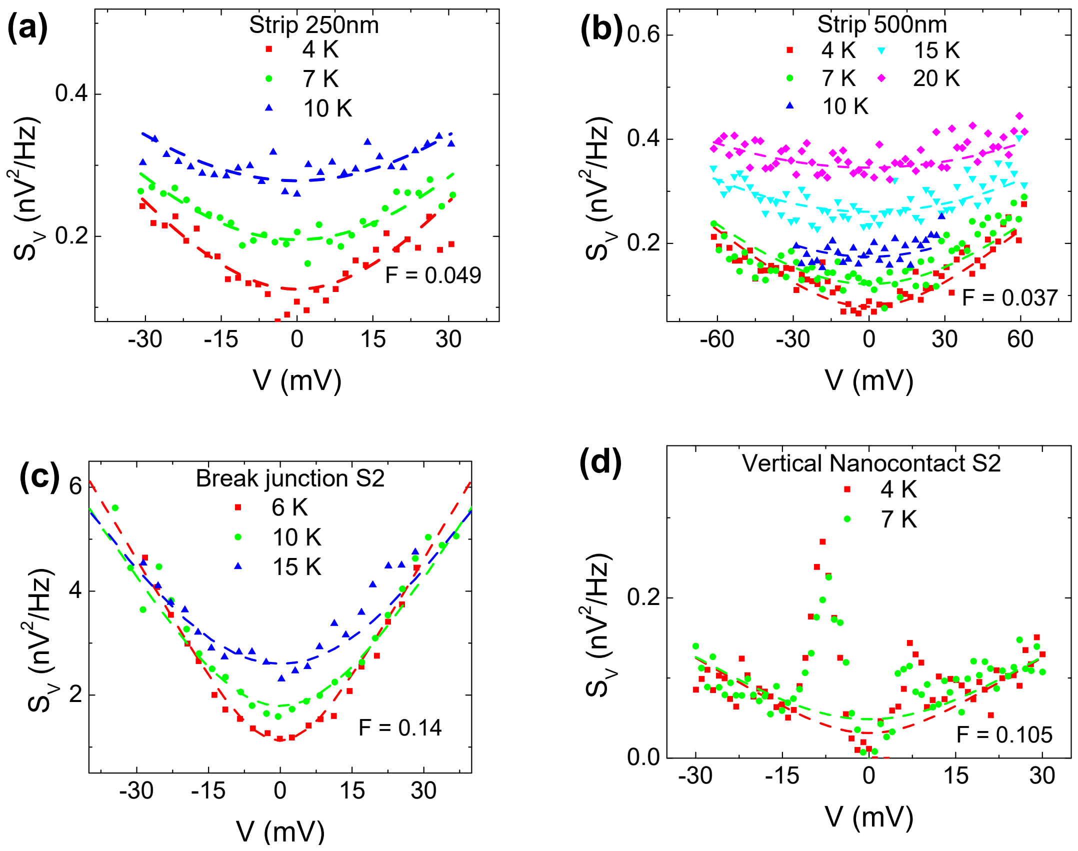

Figure S8: S_V as function a V for four additional nano-devices. The dashed lines represent shot and Johnson noise given by Eq. (S8) with the corresponding Fano factors.

Noise measurement on a control gold nanowire

We validated our measurement approach and analysis by studying noise produced by a nanowire fabricated from gold, which is a conventional Fermi liquid metal, using the same setup as in our studies of SIO. The fabrication procedure is similar to that for SIO metallic junctions, except that a sapphire chip with pre-patterned gold electrodes is used as the substrate. First, a nanowire with 60 nm width is defined by e-beam lithography and Cr(1.5)/Au(5) is deposited by thermal evaporation. Second, two Ti(2)/Au(50) electrodes separated by the $1\mu m$ gap are thermally evaporated.

Resistance versus temperature measurement (Fig.S9(a)) confirms good Ohmic contact and Fermi liquid behavior, whereas the noise spectra (Fig.S9(b)) show much smaller EMI compared with $SrIrO_3$ break junctions (Fig.3b), due to much smaller resistance. "On the other hand, fluctuations in the noise spectra are more significant due to small voltage noise. To improve measurement statistics, we choose a larger frequency window (100 - 400 kHz) to determine the white noise. At 4.5 K, the shot noise produced by the nanowire shows good match with Eq.(S9) at small bias voltage, giving F = 1/3, except for a few outlier points caused by EMI spikes. At large bias, shot noise exhibits a nonlinear bias dependence consistent with $S_V \propto V^{0.4}$ as predicted for the effects of electron-phonon scattering.[7] At higher temperature, $F = 1/3$ regime cannot be observed due to increased thermal broadening and enhanced effects of electron-phonon scattering.

References

(1) Nie, Y. F.; King, P.; Kim, C.; Uchida, M.; Wei, H.; Faeth, B. D.; Ruf, J.; Ruff, J.; Xie, L.; Pan, X.; others Interplay of spin-orbit interactions, dimensionality, and octahedral rotations in semimetallic SrIrO 3. *Physical review letters* **2015**, *114*, 016401.

(2) Sen, K.; Fuchs, D.; Heid, R.; Kleindienst, K.; Wolff, K.; Schmalian, J.; Le Tacon, M. Strange semimetal dynamics in SrIrO3. *Nature Communications* **2020**, *11*, 4270.

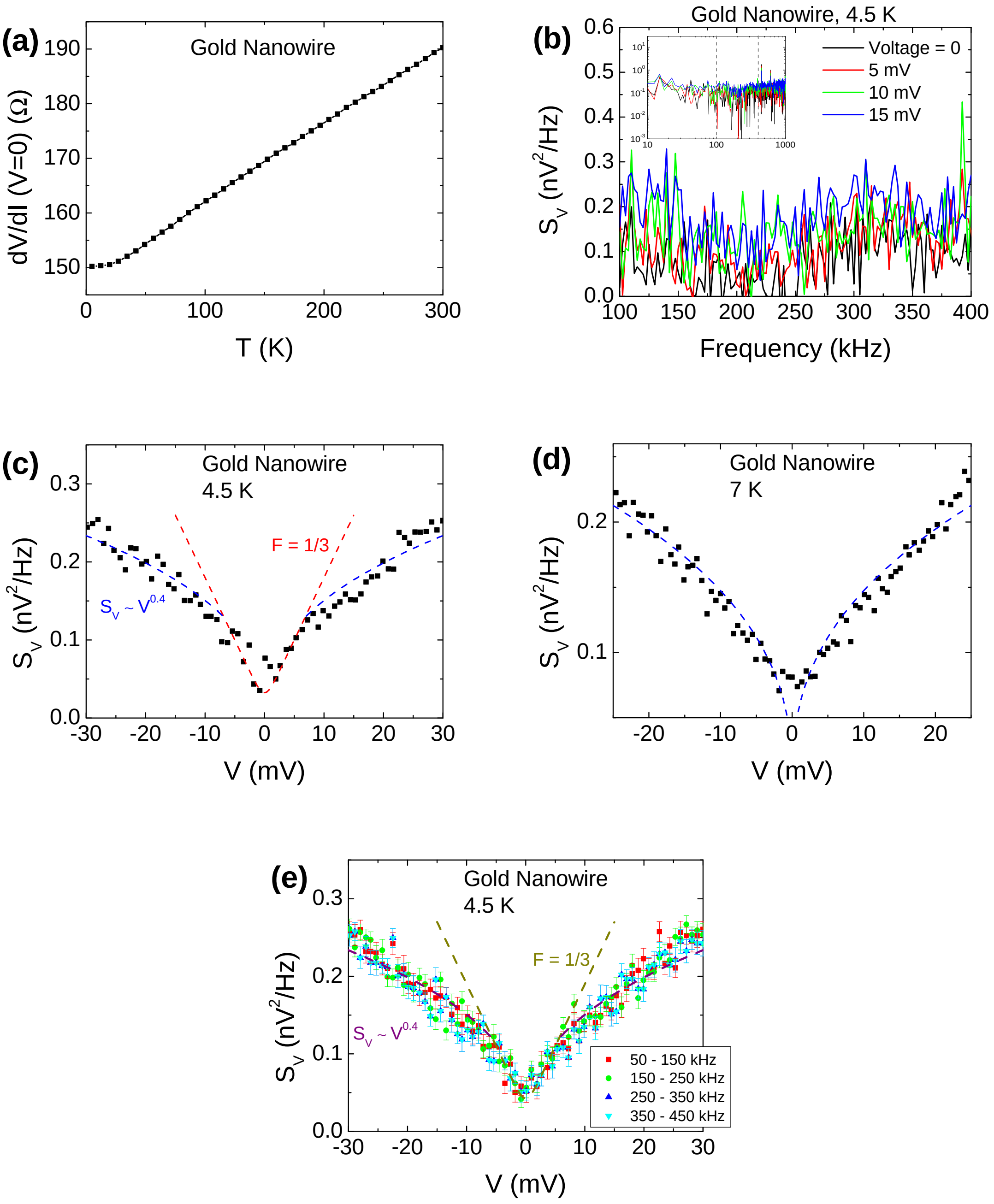

Figure S9: (a) Resistance of a 1 μm-long gold nanowire as a function of temperature. (b) Noise spectra of the nanowire under bias. (c),(d) S_V as a function a V at (c) 4.5 K and (d) 7 K. Red dashed line represents Johnson plus shot noise with $F = 1/3$, and blue dashed line represents shot noise vs bias in the electron-phonon scattering regime. (e) Shot noise of gold nanowire at 4.5 K calculated using different frequency intervals.

(3) Manca, N.; Groenendijk, D. J.; Pallecchi, I.; Autieri, C.; Tang, L. M.; Telesio, F.; Mattoni, G.; McCollam, A.; Picozzi, S.; Caviglia, A. D. Balanced electron-hole transport in spin-orbit semimetal SrIrO 3 heterostructures. *Physical Review B* **2018**, *97*, 081105.

(4) Yamasaki, A.; Fujiwara, H.; Tachibana, S.; Iwasaki, D.; Higashino, Y.; Yoshimi, C.; Nakagawa, K.; Nakatani, Y.; Yamagami, K.; Aratani, H.; others Three-dimensional electronic structures and the metal-insulator transition in Ruddlesden-Popper iridates. *Physical Review B* **2016**, *94*, 115103.

(5) Groenendijk, D. J.; Manca, N.; de Bruijckere, J.; Monteiro, A. M. R.; Gaudenzi, R.; van der Zant, H. S.; Caviglia, A. D. Anisotropic magnetoresistance in spin–orbit semimetal SrIrO 3. *The European Physical Journal Plus* **2020**, *135*, 627.

(6) Beenakker, C.; Büttiker, M. Suppression of shot noise in metallic diffusive conductors. *Physical Review B* **1992**, *46*, 1889.

(7) Nagaev, K. On the shot noise in dirty metal contacts. *Physics Letters A* **1992**, *169*, 103–107.

(8) Nagaev, K. Influence of electron-electron scattering on shot noise in diffusive contacts. *Physical Review B* **1995**, *52*, 4740.

(9) Chen, L.; Lowder, D. T.; Bakali, E.; Andrews, A. M.; Schrenk, W.; Waas, M.; Svagera, R.; Eguchi, G.; Prochaska, L.; Wang, Y.; others Shot noise in a strange metal. *Science* **2023**, *382*, 907–911.

(10) Wellstood, F.; Urbina, C.; Clarke, J. Hot-electron effects in metals. *Physical Review B* **1994**, *49*, 5942.
